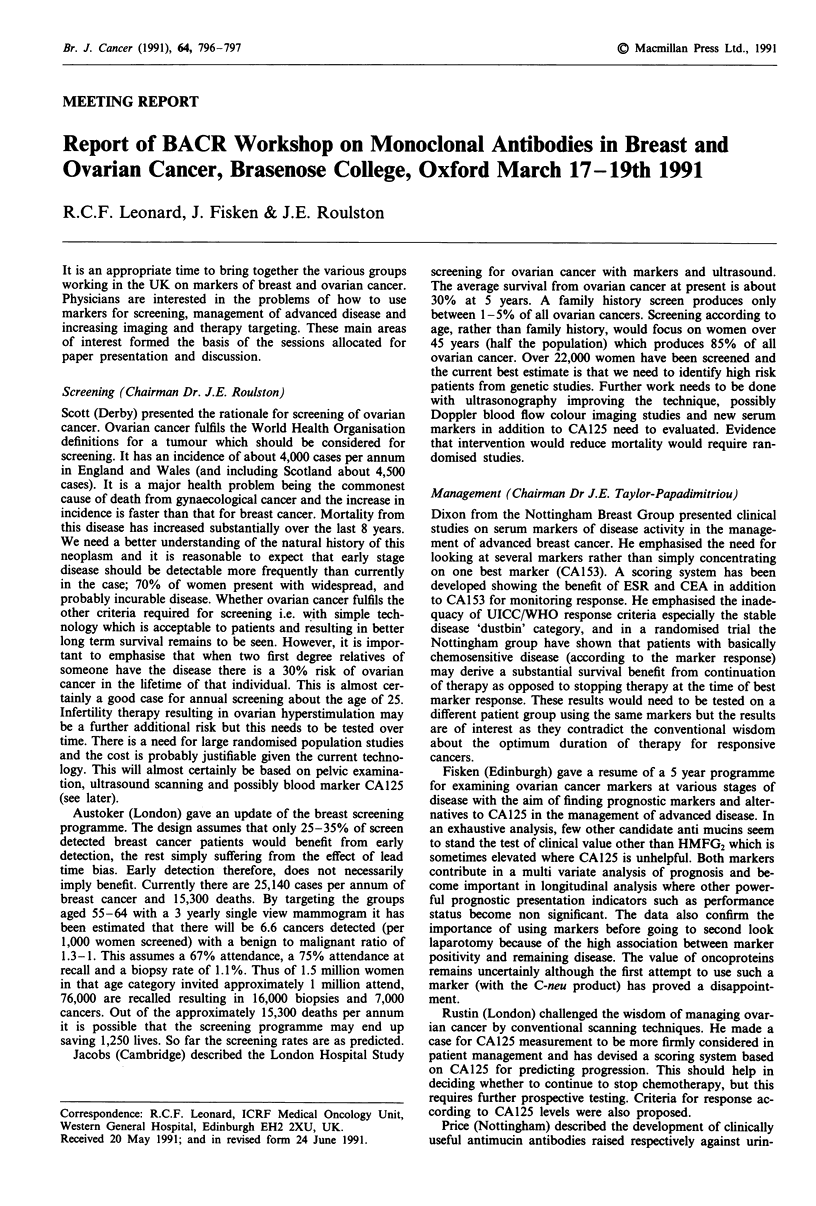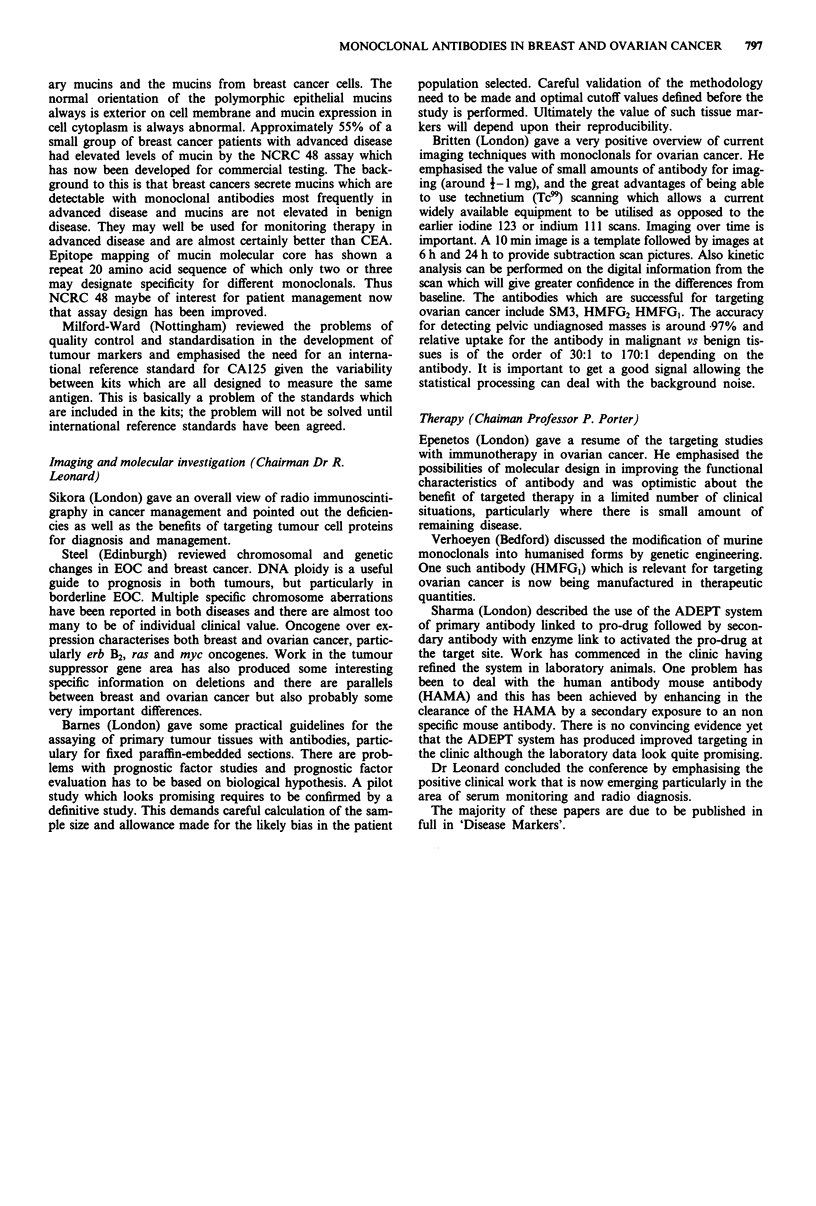# Report of BACR workshop on monoclonal antibodies in breast and ovarian cancer, Brasenose College, Oxford March 17-19th 1991.

**DOI:** 10.1038/bjc.1991.402

**Published:** 1991-10

**Authors:** R. C. Leonard, J. Fisken, J. E. Roulston

**Affiliations:** ICRF Medical Oncology Unit, Western General Hospital, Edinburgh, UK.


Br. J. Cancer (1991), 64, 796-797 © Macmillan Press Ltd., 1991

MEETING REPORT

### Report of BACR Workshop on Monoclonal Antibodies in Breast and Ovarian Cancer, Brasenose College, Oxford March 17-19th 1991

R.C.F. Leonard, J. Fisken & J.E. Roulston

It is an appropriate time to bring together the various groups working in the UK on markers of breast and ovarian cancer. Physicians are interested in the problems of how to use markers for screening, management of advanced disease and increasing imaging and therapy targeting. These main areas of interest formed the basis of the sessions allocated for paper presentation and discussion.

*Screening (Chairman Dr. J.E. Roulston)*

Scott (Derby) presented the rationale for screening of ovarian cancer. Ovarian cancer fulfils the World Health Organisation definitions for a tumour which should be considered for screening. It has an incidence of about 4,000 cases per annum in England and Wales (and including Scotland about 4,500 cases). It is a major health problem being the commonest cause of death from gynaecological cancer and the increase in incidence is faster than that for breast cancer. Mortality from this disease has increased substantially over the last 8 years. We need a better understanding of the natural history of this neoplasm and it is reasonable to expect that early stage disease should be detectable more frequently than currently in the case; 70% of women present with widespread, and probably incurable disease. Whether ovarian cancer fulfils the other criteria required for screening i.e. with simple technology which is acceptable to patients and resulting in better long term survival remains to be seen. However, it is important to emphasise that when two first degree relatives of someone have the disease there is a 30% risk of ovarian cancer in the lifetime of that individual. This is almost certainly a good case for annual screening about the age of 25. Infertility therapy resulting in ovarian hyperstimulation may be a further additional risk but this needs to be tested over time. There is a need for large randomised population studies and the cost is probably justifiable given the current technology. This will almost certainly be based on pelvic examination, ultrasound scanning and possibly blood marker CA125 (see later).

Austoker (London) gave an update of the breast screening programme. The design assumes that only 25-35% of screen detected breast cancer patients would benefit from early detection, the rest simply suffering from the effect of lead time bias. Early detection therefore, does not necessarily imply benefit. Currently there are 25,140 cases per annum of breast cancer and 15,300 deaths. By targeting the groups aged 55-64 with a 3 yearly single view mammogram it has been estimated that there will be 6.6 cancers detected (per 1,000 women screened) with a benign to malignant ratio of 1.3-1. This assumes a 67% attendance, a 75% attendance at recall and a biopsy rate of 1.1%. Thus of 1.5 million women in that age category invited approximately 1 million attend, 76,000 are recalled resulting in 16,000 biopsies and 7,000 cancers. Out of the approximately 15,300 deaths per annum it is possible that the screening programme may end up saving 1,250 lives. So far the screening rates are as predicted.

Jacobs (Cambridge) described the London Hospital Study screening for ovarian cancer with markers and ultrasound. The average survival from ovarian cancer at present is about 30% at 5 years. A family history screen produces only between 1-5% of all ovarian cancers. Screening according to age, rather than family history, would focus on women over 45 years (half the population) which produces 85% of all ovarian cancer. Over 22,000 women have been screened and the current best estimate is that we need to identify high risk patients from genetic studies. Further work needs to be done with ultrasonography improving the technique, possibly Doppler blood flow colour imaging studies and new serum markers in addition to CA125 need to evaluated. Evidence that intervention would reduce mortality would require randomised studies.

*Management (Chairman Dr J.E. Taylor-Papadimitriou)*

Dixon from the Nottingham Breast Group presented clinical studies on serum markers of disease activity in the management of advanced breast cancer. He emphasised the need for looking at several markers rather than simply concentrating on one best marker (CA153). A scoring system has been developed showing the benefit of ESR and CEA in addition to CA153 for monitoring response. He emphasised the inadequacy of UICC/WHO response criteria especially the stable disease 'dustbin' category, and in a randomised trial the Nottingham group have shown that patients with basically chemosensitive disease (according to the marker response) may derive a substantial survival benefit from continuation of therapy as opposed to stopping therapy at the time of best marker response. These results would need to be tested on a different patient group using the same markers but the results are of interest as they contradict the conventional wisdom about the optimum duration of therapy for responsive cancers.

Fisken (Edinburgh) gave a resume of a 5 year programme for examining ovarian cancer markers at various stages of disease with the aim of finding prognostic markers and alternatives to CA125 in the management of advanced disease. In an exhaustive analysis, few other candidate anti mucins seem to stand the test of clinical value other than $HMFG_2$ which is sometimes elevated where CA125 is unhelpful. Both markers contribute in a multi variate analysis of prognosis and become important in longitudinal analysis where other powerful prognostic presentation indicators such as performance status become non significant. The data also confirm the importance of using markers before going to second look laparotomy because of the high association between marker positivity and remaining disease. The value of oncoproteins remains uncertainly although the first attempt to use such a marker (with the C-*neu* product) has proved a disappointment.

Rustin (London) challenged the wisdom of managing ovarian cancer by conventional scanning techniques. He made a case for CA125 measurement to be more firmly considered in patient management and has devised a scoring system based on CA125 for predicting progression. This should help in deciding whether to continue to stop chemotherapy, but this requires further prospective testing. Criteria for response according to CA125 levels were also proposed.

Price (Nottingham) described the development of clinically useful antimucin antibodies raised respectively against urin-

Correspondence: R.C.F. Leonard, ICRF Medical Oncology Unit, Western General Hospital, Edinburgh EH2 2XU, UK.
Received 20 May 1991; and in revised form 24 June 1991.

ary mucins and the mucins from breast cancer cells. The normal orientation of the polymorphic epithelial mucins always is exterior on cell membrane and mucin expression in cell cytoplasm is always abnormal. Approximately 55% of a small group of breast cancer patients with advanced disease had elevated levels of mucin by the NCRC 48 assay which has now been developed for commercial testing. The background to this is that breast cancers secrete mucins which are detectable with monoclonal antibodies most frequently in advanced disease and mucins are not elevated in benign disease. They may well be used for monitoring therapy in advanced disease and are almost certainly better than CEA. Epitope mapping of mucin molecular core has shown a repeat 20 amino acid sequence of which only two or three may designate specificity for different monoclonals. Thus NCRC 48 maybe of interest for patient management now that assay design has been improved.

Milford-Ward (Nottingham) reviewed the problems of quality control and standardisation in the development of tumour markers and emphasised the need for an international reference standard for CA125 given the variability between kits which are all designed to measure the same antigen. This is basically a problem of the standards which are included in the kits; the problem will not be solved until international reference standards have been agreed.

*Imaging and molecular investigation (Chairman Dr R. Leonard)*

Sikora (London) gave an overall view of radio immunoscintigraphy in cancer management and pointed out the deficiencies as well as the benefits of targeting tumour cell proteins for diagnosis and management.

Steel (Edinburgh) reviewed chromosomal and genetic changes in EOC and breast cancer. DNA ploidy is a useful guide to prognosis in both tumours, but particularly in borderline EOC. Multiple specific chromosome aberrations have been reported in both diseases and there are almost too many to be of individual clinical value. Oncogene over expression characterises both breast and ovarian cancer, particularly *erb* $B_2$, *ras* and *myc* oncogenes. Work in the tumour suppressor gene area has also produced some interesting specific information on deletions and there are parallels between breast and ovarian cancer but also probably some very important differences.

Barnes (London) gave some practical guidelines for the assaying of primary tumour tissues with antibodies, particulary for fixed paraffin-embedded sections. There are problems with prognostic factor studies and prognostic factor evaluation has to be based on biological hypothesis. A pilot study which looks promising requires to be confirmed by a definitive study. This demands careful calculation of the sample size and allowance made for the likely bias in the patient population selected. Careful validation of the methodology need to be made and optimal cutoff values defined before the study is performed. Ultimately the value of such tissue markers will depend upon their reproducibility.

Britten (London) gave a very positive overview of current imaging techniques with monoclonals for ovarian cancer. He emphasised the value of small amounts of antibody for imaging (around $\frac{1}{2}$–1 mg), and the great advantages of being able to use technetium ($Tc^{99}$) scanning which allows a current widely available equipment to be utilised as opposed to the earlier iodine 123 or indium 111 scans. Imaging over time is important. A 10 min image is a template followed by images at 6 h and 24 h to provide subtraction scan pictures. Also kinetic analysis can be performed on the digital information from the scan which will give greater confidence in the differences from baseline. The antibodies which are successful for targeting ovarian cancer include SM3, $HMFG_2$ $HMFG_1$. The accuracy for detecting pelvic undiagnosed masses is around 97% and relative uptake for the antibody in malignant *vs* benign tissues is of the order of 30:1 to 170:1 depending on the antibody. It is important to get a good signal allowing the statistical processing can deal with the background noise.

*Therapy (Chaiman Professor P. Porter)*

Epenetos (London) gave a resume of the targeting studies with immunotherapy in ovarian cancer. He emphasised the possibilities of molecular design in improving the functional characteristics of antibody and was optimistic about the benefit of targeted therapy in a limited number of clinical situations, particularly where there is small amount of remaining disease.

Verhoeyen (Bedford) discussed the modification of murine monoclonals into humanised forms by genetic engineering. One such antibody ($HMFG_1$) which is relevant for targeting ovarian cancer is now being manufactured in therapeutic quantities.

Sharma (London) described the use of the ADEPT system of primary antibody linked to pro-drug followed by secondary antibody with enzyme link to activated the pro-drug at the target site. Work has commenced in the clinic having refined the system in laboratory animals. One problem has been to deal with the human antibody mouse antibody (HAMA) and this has been achieved by enhancing in the clearance of the HAMA by a secondary exposure to an non specific mouse antibody. There is no convincing evidence yet that the ADEPT system has produced improved targeting in the clinic although the laboratory data look quite promising.

Dr Leonard concluded the conference by emphasising the positive clinical work that is now emerging particularly in the area of serum monitoring and radio diagnosis.

The majority of these papers are due to be published in full in 'Disease Markers'.